# Surgical Cases (So-Called), Cured Homœopathically

**Published:** 1893-01

**Authors:** W. E. Ledyard

**Affiliations:** San Francisco, Cal.


					﻿TWO SUCCESSFUL HOMOEOPATHIC SURGICAL
OPERATIONS.
J. H. Allen, M. D., Logansport, Ind.
Case A.—Mrs. C., age fifty-one, large and fleshy, of a bilious
temperament, subject to rheumatism, and catarrhal colds of the
head and lungs; has family history of psora. Has always been
treated homceopathically. Six years ago I removed a large
tumor from the left scapular region, from which she suffered
much pain in using the arm. After the removal of the growth,
which was a large one, the wound healed kindly and I dis-
charged her in a few weeks apparently cured. I did not see nor
hear from her until one year ago, when she presented herself for
another operation, a tumor of a similar nature, which had for
the past two years been growing on the left shoulder about three
inches from the seat of the former growth, but more anterior,
extending toward the neck. I made a diagnosis of the growth
similar to the one I had operated upon. When I inquired why
she had not applied for treatment sooner, she replied that she
knew that nothing but an operation would be of any benefit to
her, and as long as she was suffering no inconvenience from it
she thought it best not to interfere. It was now causing her
considerable pain and annoyance and wished an operation. I
should think it would weigh about five pounds. Within the
last two months it has grown rapidly and, in appearance, it
looked as if it might become a large growth. I assured her
that we would not have to operate this time and prescribed
Psorinum10m, Fincke, followed by no medicine for two months. I
do not remember now the symptoms I prescribed it on, but at the
end of this time there had developed the following symptoms:
feet, hands, and face bloated in the morning. No pain in the
tumor now, but can see no change in its size or shape.
She perspires freely under the arms, which has a very offensive
odor and which rots her clothing. The feet also perspire a great
deal, the odor from which is horribly offensive. Has much raw-
ness between the toes, and, for weeks at a time, the skin peels
off them.
Silicea'” was given, and during the period of ninety days,
when all the above symptoms disappeared together with the
growth. The rapidity with which this growth disappeared
under the profound action of this highly potentized Silicea was
truly remarkable; it was as much of a surprise to myself as the
patient.
What a pleasure it is to cure one of these cases without the
aid of the knife. We have in this case a double cure ; not only
have we cured the abnormal growth, but we have removed that
underlying principle that predisposed her from ever in the
future being a tumor-producing patient.
Case B.—Mrs. B-------, age twenty-seven, brunette, married
eightyears, has one child, age seven, family history good, father
and mother living and well. She has had almost perfect health up
to three years ago, when she began to suffer with severe pains in
the uterus during the menses and almost constant neuralgia of the
ovaries during inter-menstrual period. For the past year she
suffered more or less with chills which were very severe, com-
ing on early in the morning and lasting for an hour or more,
followed by fever which lasted until about two o’clock in the
morning, when she would break into a profuse sweat. She
seldom slept until the sweating began. She has emaciated rapidly
within the past six months of her illness. Her weight has de-
creased in the past two months thirty pounds. During the
past two months she has been confined to her bed and treated
for lung trouble, though she has neither cough, soreness, or any
pain in the lungs, nor is there any history of a lung trouble in
the family. But on a careful examination of her case I find a
cancer of the uterus in an advanced stage and that of a sycotic
nature. My opinion was confirmed by Dr. E. R. Sawyer, of
Kokomo, Ind. The whole right anterior half of the body of
uterus was involved, it also extended, in the form of a band,
around the rectum, which had begun to ulcerate and is discharg-
ing at each stool bloody pus and mucus. The bladder is being
infringed upon somewhat, which causes urination to be more
frequent and painful. The chills come every day, followed by
fever and profuse and debiltating sweats, for which China was
prescribed. The menses have not made their appearance for
three months. Previous to the suppression of the menses she
had been treated for a profuse acrid leucorrhoea by medicated
injections. The urethral orifice is very much swollen, vagina
sensitive, ovaries somewhat enlarged and sensitive to pressure.
There is a slight watery discharge from the uterus, which pro-
duces intense pruritus, and which is relieved by frequent bathing,
and has an odor of decayed fish. Medorrinum was prescribed
in the CM potency, followed by no medicine for two months.
Marked improvement followed this prescription ; in one week all
her pains had ceased. The ovaries are less sensitive and the
symptoms much better in every way. Menstruation came On
at the end of the second week after this prescription—though
painful, it seems quite normal otherwise. The pruritus has
ceased and she cannot detect that peculiar fishy odor nearly as
plainly. Chills do not come on nearly as often ; for the past
four days has had none, which is the first time they have re-
mained away so long for six months. At the end of the eighth
week I gave another powder, as she was not as well. From
that time on she rapidly grew better. The face grows less
pinched and the color better. Her eyes have a less hollow look,
and the dark circles are leaving them. The sweats are much
less, and she sleeps all night, feeling less exhausted in the morn-
ing. No more medicine was given for sixty days, when a severe
diarrhoea set in calling for Podophyllum, which was given in
water, followed by no medicine for one week, when Conium
was prescribed, based upon the symptoms of the mammary gland
during the menstrual period.
For the past two months she has done all her own house
work, and is improving every day. On examination I find the
growth has disappeared and the hypertrophied walls of the
uterus have almost resumed their normal condition. A slight
thickening remains in the uterine walls, but the tenderness and
soreness have all disappeared. I prescribed for her before I left
for this meeting what I considered in her case a good prescrip-
tion, NO MEDICINE.
ADDITIONAL REPORT OF BOARD OF CENSORS.
James Ferrier, M. D., of New York, Alfred J. Norman,
M. D., of Rochester, and Marvin A. Custis, M. D., of Wash-
ington, D. C., were elected to junior membership, the Secretary
casting the ballot for each.
REPORT OF COMMITTEE ON VACCINATION.
In considering the resolutions on Vaccination, your Commit-
tee deems the first one of so great importance to every member
of this Association that we would suggest that it be referred to
a special committee of three, whose duty it shall be to make a
general report on the subject of vaccination at the next annual
meeting.
Regarding the second resolution, your Committee unanimously
recommends its adoption.
H. Hitchcock, Chairman.
A. R. Morgan,
W. P. Defriez,
The second resolution, as follows, was adopted :
Resolved, That compulsory vaccination is unjustifiable and
contrary to the rights and privileges of the people.

				

