# Correction

**Published:** 1893-02

**Authors:** 


Correction.—In the January number, at page 19, line twenty-two from the top for S. L., read Syphilinum$^{mm}$.

